# The Dependence of the Burning Process and Ignition Temperature of a Lithium Cell on Its State of Charge

**DOI:** 10.3390/s23020753

**Published:** 2023-01-09

**Authors:** Andrzej Erd, Tomasz Ciszewski

**Affiliations:** Faculty of Transport, Electrical Engineering and Computer Science, Kazimierz Pulaski University of Technology and Humanities in Radom, 26-600 Radom, Poland

**Keywords:** cell ignition, state of capacity, battery management systems, thermal management systems

## Abstract

Batteries and energy stores built with lithium-ion cells are potentially dangerous and can cause fires that are difficult to extinguish. Reducing the intensity of the fires and extending the time of their development may be of great importance for improving safety. The aim of this work is to examine the influence of the state of charge (SOC) of a cell on susceptibility to ignition, and to analyze the course of the burning process. For this purpose, a special measuring station was built, where ignition was initiated and the course of combustion was observed. During the measurements, energy was supplied by heating a cell from the outside with a resistance heater while at the same time thermally insulating the cell from the environment. The measures of the course of the fire were the amount of energy supplied to the cell before ignition and the temperature changes during the fire. The tests proved the existence of significant differences in the amount of energy causing the ignition of cells. These differences result from changes in the SOC. Quantitative results are presented. The existence of differences in susceptibility to ignition can be used to change the construction of control algorithms for battery management systems (BMSs).

## 1. Introduction

The observed intensive development of electric vehicles is largely caused by the concern to reduce the burden on the environment caused by the products of combustion of organic materials. This concern intensifies the development of methods and means of storing electricity. The purpose of energy storage is only primarily to use it for traction purposes. Much greater needs for storage arise in connection with the development of energy related to renewable sources, in particular with photovoltaic farms. Having energy storage facilities stabilizes the power supply conditions, reduces the load on transmission lines and allows for reductions in current energy costs. However, the construction of batteries and storage facilities requires a huge number of individual cells [1,2]. For a variety of reasons, batteries are built from individual smaller cells, which, however, have many disadvantages.

The main disadvantages are a long safe charging time and low resistance to irregularities in the charging process [3,4], which may result in self-ignition or even explosion [5,6]. In the currently produced lithium cells, if the limit parameters are exceeded, a dangerous process of thermal runaway may develop [7,8]. In the papers [9,10,11], the course of the thermal runaway process for a fully charged cell was considered. In particular, the course of electrochemical processes and their impact on the level of heat and oxygen emissions were analyzed.

Existing threats are being mitigated in many ways [12,13]. One of these is the construction of batteries in such a way as to limit heat transfer between cells. This is achieved by using spacers made of various materials that conduct heat poorly. An analysis of this method is included in [14]. The use of phase change materials (PCMs) is an approach that allows the ignition of a cell to be delayed due to its excessive heating, and is widely used [15,16]. A comprehensive overview of cooling technology is contained in [17,18]. The combination of PCM with cooling systems and the construction of appropriate heat sinks allows for simplification cooling systems [19]. However, it has been noted that the use of certain cooling techniques can be dangerous [20]. A more general approach is the construction of thermal management systems (TMSs) [21,22,23] and battery management systems (BMSs) [24,25,26,27], which cover all issues of energy and heat transmission in the battery [23,28]. A Special Issue of the journal Energies [29] is devoted to this topic. The article [30] describes the research on the influence of the state of charge (SOC) on the safety of UN38 cells in air transport.

The main purpose of the presented research is to determine whether there are significant differences in combustible properties between cells of the same type depending on the state of charge of the cell. The subject of the research is determining the influence of the state of charge (SOC) of a cell on the course of combustion and the ignition temperature. An experimental method was adopted that allowed the ignition temperature to be explicitly determined. Among the many possible factors that may trigger the initiation of a fire, the supply of energy in the form of heat was assumed. This is important because this factor can affect the cell even in the off state and regardless of the system in which the cell is built. In order to investigate the relationship between the SOC and the course of the cell fire, experiments were planned and conducted on a series of identical cells, for which the only differentiating factor was their state of charge. The second objective of the study was to determine the parameters of the fire course in the form of the maximum temperature and the temperature increase rate, also depending on the SOC. The expected result was that the characteristics of temperature change as a function of time, assuming heating with the same heating power. The preliminary estimation of the amount of energy released during fires was adopted as a conditional goal.

The method of organization of the research that was adopted is similar to that used in [31]. Chapter 2: Materials and Methods describes the tested cell and the preliminary tests carried out using thermography, the results of which are included in Chapter 3.1. The following sections describe various aspects of the test stand (2.1) which allow the performance of high-frequency measurements of both electrical parameters and temperatures. The test stand’s most important features are the averaging of measurements on an ongoing basis and registration in data files. Measurements were made using the LabView system. Section 2.2 describes the virtual instruments created for the experiment. Section 2.3 presents the measuring block and the cell module, the construction of which provides thermal insulation from the environment and the measurement of heat released during a cell fire. For this purpose, apart from the cell temperature, the temperatures of sand, water and air in the measuring chamber were measured. Section 2.4 briefly describes the measurement cycle.

Chapter 3 describes the results achieved, successively, for various states of charge: 0%, 30%, 50% and 100%, as well as for cells discharged in a state lower than the limit and overcharged.

During the research, it was noted that it is possible to create cell temperature characteristics depending on the amount of energy supplied to the cell module as an independent variable. These characteristics are more general than the dependence on time. These characteristics are also included in Section 3.2.7. In addition, estimations of the amount of energy released by the burning cells as a function of SOC were made. Chapter 4 contains a discussion of the results achieved and the possibilities of improving the metrological properties noticed during the experimental tests, and a plan for the next stage of research. Chapter 5 presents more general conclusions that may be an inspiration for further research for other teams and cell manufacturers.

## 2. Materials and Methods

The research presented in this article was conducted on lithium-ion cells made using INR technology. The declared maximum current was 40 A. Table 1 lists the specifications of the cell.

The cells accepted for testing came from the same production batch. After delivery, the no-load voltage was measured for all cells and no differences greater than 0.002 V were detected. Therefore, they were assumed to be identical. 

A special test stand was constructed to carry out the tests, the various aspects of which are described in Section 2.1, Section 2.2 and Section 2.3. The characteristics of the stand were as follows:

- Possibly full thermal insulation from the environment of the cell subject to testing;
- Accurate measurement of cell temperature in a wide range;
- Parallel cell temperature measurements by two different measurement systems and sensors;
- Temperature measurements of cooling agents and elements of the measuring block;
- Mass measurements of the measuring block elements before and after the next test;
- Registration of all parameters measured automatically with saving to .xlsx files;
- High accuracy of measurements of both electrical parameters and temperatures, due to the use of high-quality measurement LabView modules;
- Averaging a series of measurements, e.g., 100 with a frequency of 10 kHz and accepting the result as a point measurement in order to eliminate noise;
- The ability to charge and unload the cells in any way due to the construction of one’s own virtual devices;
- High repeatability of the test run.

The research consisted of two stages. In the initial stage, an experiment was prepared that involved placing 3 elements next to each other: a cell charged to the maximum, a cylindrical heater in the shape of a cell and a second cell discharged to the minimum voltage. After the heater was turned on, the temperature was recorded using RTD sensors placed on the cells and the heater. After heating the cells to about 60 degrees Celsius, it was found that the temperature difference is a few degrees, but the result is strongly dependent on where the sensor is attached to the cell; therefore, thermal imaging measurements were carried out. Measurements were made using a VIGO 50 thermal imaging camera and developed using the Therm program—Vigo System authorized by the camera manufacturer. The tests were carried out for the same set of elements as in the case of measurements using RTD sensors. The measurement results are included in Section 3.1.

**Figure 1:**
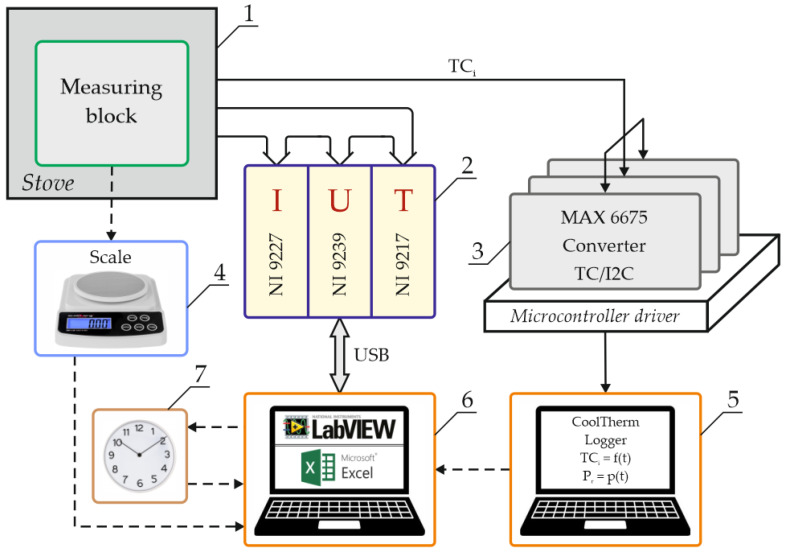
Scheme of the test stand. Number 1—measuring block shown separately in Figure 3; 2—LabView cassette for cyclic measurements of electrical parameters and current temperatures; 3—microcontroller for temperature measurements using thermocouples; 4—scale for measuring the mass of elements included in the measuring block; 5—computer archiving data from thermocouples; 6—computer archiving data from the LabView cassette; 7—internal clock in the computer.

This configuration of the measuring system was interesting because of the possibility of real-time observation of temperature changes over the entire surface of the cell. In this measurement, the amount of energy supplied to the heater was known, but it was not possible to determine what part of it went to the cells. In addition, there was no certainty whether the heat transport, which was largely carried out by conduction on small contact surfaces of the cell walls and the heater, was completely identical for both cells. It was also necessary to ensure the safety of both the observer and the camera. Therefore, it was impossible to heat the cell to ignition-threatening temperatures, i.e., higher than those recommended by the manufacturer, without the use of external covers. A decision was made to build a stand that would give the possibility of obtaining repeatable results, with a wide range of energy supplied to the cell and guaranteeing safety in the event of a cell explosion. A special test stand, presented in Figure 1, and a set of virtual instruments based on the LabView system were developed to carry out the tests.

### 2.1. Test Stand

The research presented in this article required the cell to be ignited, which in many cases resulted in a violent ignition. Therefore, in order to protect the cell, a sterilization stove was used as housing. The stove provided thermal insulation from the environment, allowed for controlling the moment of emptying the stand of exhaust fumes and, above all, protected personnel against the effects of cell explosions. The internal joints of the stove walls were sealed and a pressure sensor was installed inside the chamber. A measuring block was placed inside the furnace before each measurement. 

Measurement results obtained by LabView modules were saved directly in files in Excel format. In addition, a set of thermocouples and a pressure sensor in the furnace were attached to the measuring block. Therefore, a microprocessor controller was constructed that allows the reading of data from three MAX6675 systems. These systems have compensation for the cold end of the thermocouple and allow for relatively fast temperature measurements in a wide range.

Data from the thermocouples were saved separately to text files (in the second computer). In addition, the current timestamp was saved in the files, allowing for synchronization with files from LabView. Synchronization took place after the end of the experiment. The test stand was also equipped with a scale allowing the measurement of the mass of individual elements in the phases before and after the experiment.

### 2.2. Virtual Instruments

The LabView system was used for measurement purposes. The essence of LabView is the ability to build measurement applications using one’s own hardware modules. The software part of the application is created by the user. Programming is done using a special graphical language representing the flow of signals.

Outside the furnace was a cDAQ 9174 cassette with LabView modules. This cassette provided the possibility of ongoing communication with a personal computer via a USB interface. Modules installed in the cassette:

- NI-9239—4 Channel Voltage Input Module 0–5 V resolution-24 bit.
- NI-9217—4 Channel Temperature Input Module RTD resolution-24 bit.
- NI-9227—4 Channel Current Input Module 0–5 A resolution-24 bit.

Maximum sample rate for all channels was 50 kS/s per channel. Using these modules, it is possible to build virtual instruments that act as data recorders, in particular of electrical power and temperature.

The following virtual instruments were developed for research purposes:

- The VI1 cell charging system, which was equipped with a 4-channel voltage and current measurement with current recording in all channels separately. The VI1 system had the ability to connect and disconnect a 4-channel power supply with current and voltage stabilization in each channel. The virtual device calculated the products of the current values of current and voltage, i.e., the instantaneous charging power. Multiplying the power by the length of the time quantum between measurements and adding them up resulted in the calculation of the amount of energy transferred to the cells during charging. The registration in the file included the time from the start of the measurements, voltage, current and energy, which allowed the charging characteristics of the cells to be found.
- The VI2 cell discharge system was designed to discharge the cell in a controlled way, so as to leave the desired part of the full charge in it. After giving up the assumed amount of energy, the cell was disconnected from the load. Another criterion for the completion of discharging was the assumed voltage value to which the cell was discharged. In this way, it was possible to precisely charge and discharge cells to the required SOC. Unlike with factory devices, it was possible to bypass the protections that are built into the automatic chargers’ software, such as by exceeding the maximum charging voltage or discharging the cell voltage to zero, i.e., below the level allowed by the manufacturer.
- Process recording system VI3. The purpose of building this system was to simultaneously register the state of the measuring module and the amount of energy transferred to the cell module. In order to reduce the number of modules in the cassette, an indirect method of current measurement was used during the measurements by using a resistor connected in series to the heater power supply system. This made it possible to eliminate the current measurement module from this virtual device. The current value was obtained by measuring the voltage across a resistor treated as a current shunt. The voltage was taken from the heater terminals. The product of the voltage determined in this way and the current was the instantaneous power transferred to the heater. The temperature module made it possible to determine the temperature in four points of the measuring block of the station.

The aforementioned virtual devices, VI1 and VI2, were used to prepare the cells in the desired state of charge in the subsequent phases of the experiment. An important advantage of using LabView was the ability to build in the measurement algorithm several samplings in a short time (10 samples/s) and determine the average value from them. In this way, waveforms without random fluctuations were obtained. Measurements using thermocouples went straight to the report files, without preliminary averaging. They were burdened with larger random errors, but in return the interval between successive samples was much smaller, which made it possible to detect even very short temperature peaks.

### 2.3. Measuring Block

**Figure 2:**
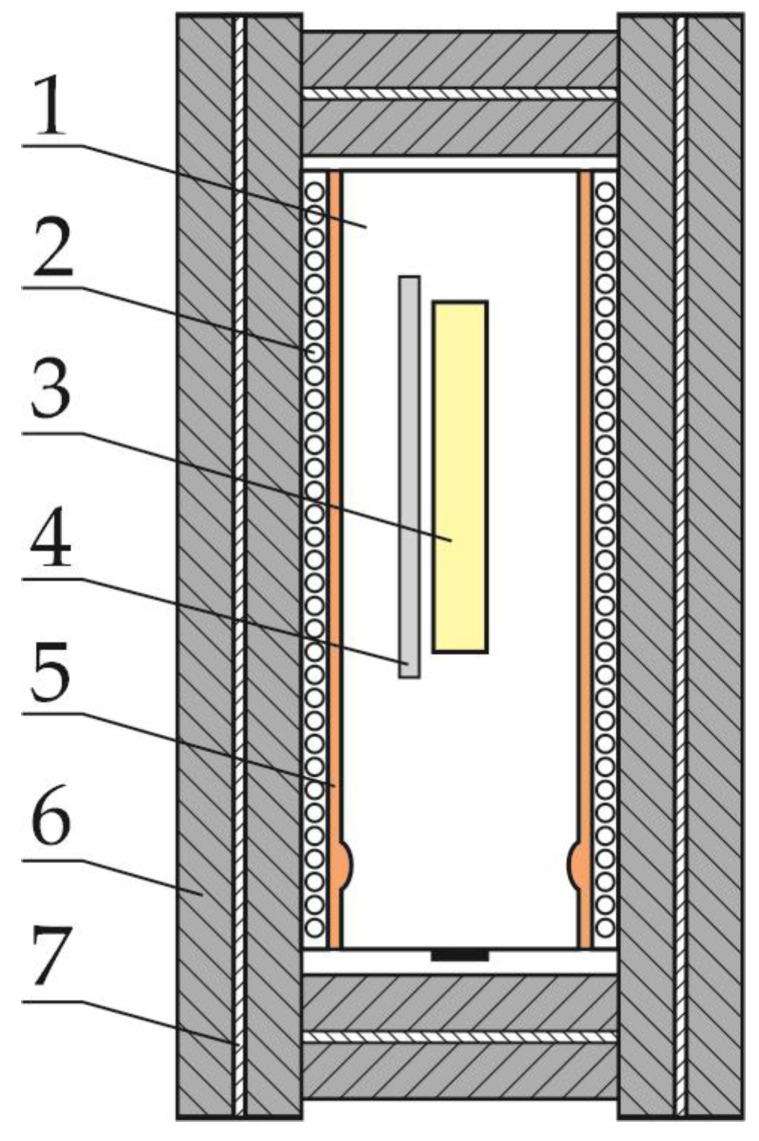
Scheme of the cell module. Number 1—cell; 2—resistant wire coil; 3—RTD Sensor; 4—thermocouple; 5—electric insulation layer; 6—fire-resistant non-woven fabric; 7—metallized fabric.

The main idea behind the construction of the measuring block consisted in achieving the best possible thermal insulation from the cell’s surroundings, while ensuring the possibility of its heating. It was also essential to be able to observe changes in the temperature of both the cell itself and its surroundings. Therefore, the cell was placed in the cell assembly shown in Figure 2. The module shown in Figure 2 contains a cell (1) on which a single-layer coil of resistance wire (2) is wound. The coil is electrically insulated from the cell with glass fiber tape (5). A thermocouple (4) and an RTD sensor (3) are inserted under the tape. Figure 2 omits wiring in the form of leads to the cell terminals, thermocouple, RTD sensor and heater power supply; thus, both the tape and the coil stabilize the position of the temperature sensors on the cell. The installation of two cell temperature sensors at the same time is intended to reduce the likelihood of missing results if one of the sensors fails. This is especially important when the temperature is greater than 500 degrees Celsius. The module cell was wrapped with two layers of fire-resistant non-woven fabric (6), between which a layer of metallized fabric (7) was placed.

This layer caused the heat to be reflected inward, i.e., reducing the emissivity of the assembly, thus reducing energy losses. Similarly, both ends of the cell were thermally insulated. Before each subsequent experiment, it was necessary to prepare another cell assembly. All assemblies were equipped with the same thermocouples and RTD sensors and were wound with the same amount of resistance wire. The cell was always placed with the positive pole down, because, as previous experience has shown, this part of the cell is less mechanically strong and the explosion usually starts from this side.

**Figure 3:**
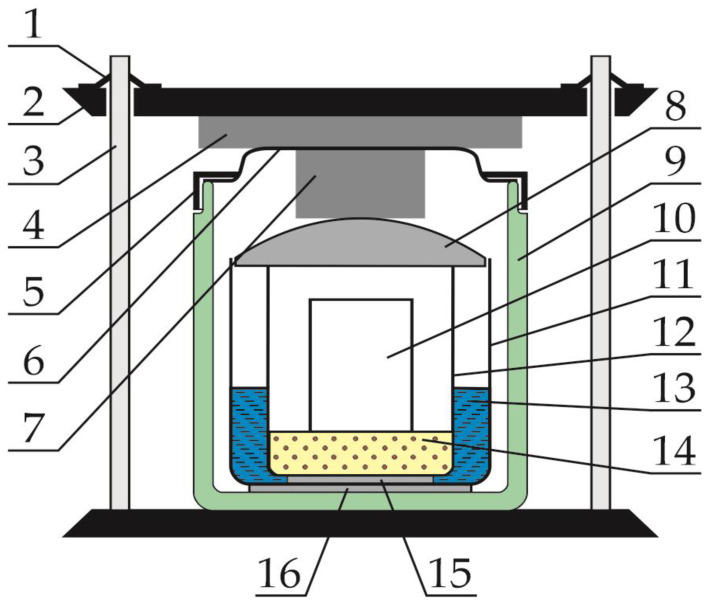
Scheme of the measuring block. Number 1—cardboard marker; 2—steel plate; 3—guide rod; 4—dumper; 5—centering ring; 6—outer cover; 7—dumper; 8—inner cover; 9—thermostatic vessel; 10—cell module; 11—intermediate vessel; 12—inner vessel; 13—cooling water; 14—quartz sand; 15—leather strips; 16—insulating pads.

The cell module that had been prepared in this way was placed in the measuring block shown in Figure 3. The cell module (10) was placed in the inner vessel (12). Quartz sand (14) was placed at the bottom of the inner vessel. In most cases, the end result of the cell ignition was an explosion. Sand was used to delay the escape of energy outside the unit and capture a large part of it. The flue gas stream coming out of the cell had a very high temperature, so the inner vessel was placed in water for cooling (13). In addition, the flue gas stream blew sand out of the interior of the inner vessel. This sand, after being lifted up, bounced off the inner cover (8) and fell back either into the inner vessel or into the water in the intermediate vessel (11) placed outside the inner vessel. In the sand, in the water and in the air above the sand in the inner vessel, RTD sensors were placed to measure the temperatures of these elements of the measuring unit. The inner vessel was largely submerged in water. Thermal insulation at the bottom between the inner and intermediate vessels was provided by natural leather strips (15). The advantage of these strips is that they are water-absorbent and therefore, when submerged, they are non-flammable and insulate the internal vessel with hot sand relatively well from the rest of the measuring unit. Since the inner vessel was made of stainless steel, the heat from the sand, the cell module and the vessel itself was transferred to the water, which in turn has a high heat capacity. It is worth noting that during the burning of highly charged cells, the exhaust gas temperature was greater than 1000 degrees Celsius. The flue gases at this temperature would have been able to melt the bottom of the inner vessel if not for the abovementioned measures to ensure cooling.

The inner vessel is covered with a glass cover with a diameter adjusted to fit in the intermediate vessel without a gap all around. All wires from the inner vessel are led outside it through two openings in the lid. In the area of the inner vessel, all wires are each separately provided with additional Teflon insulation.

The intermediate vessel was placed inside a double-walled thermostatic vessel (9) with an outer cover (6). The intermediate vessel stood on heat insulating pads (16). Due to this, the movement of heat between the vessels and the escape of heat outside the measuring block were significantly hindered. Due to the fact that the generated gases were able to lift both covers up and the cell could have been thrown out from the inside of the block, an additional heavy steel plate (2) was placed on the cover of the thermostatic vessel, which could be lifted by the pressure of gases from the inside. Between the inner cover and the outer cover of the thermostatic vessel, as well as the outer cover and the protective plate, dampers made of metal chips (4 and 7) were placed, preventing the lid and the protective plate from being hit with high acceleration. Above the plate, cardboard markers (1) were placed on the guide rods (3) to check whether the protective plate was lifted during the explosion.

### 2.4. Course of Measurements

The measurements described in this paper were carried out in several series. The previously collected results allowed for improving the apparatus and gaining experience as to the repeatability of phenomena occurring in the cells during ignition. The combination of vessels, thermal insulation, cooling, etc. was improved. For the presented paper, a series of measurements was made with identical cells, identically enclosed in the cell module. The method of execution was strictly repeatable and used the same combination of the cell module and the measuring unit. Both RTD sensors and thermocouples were used once, so the cell module was disposable. As previous experience has shown, reusing the sensors often ended up breaking them without bringing the experiment to an end. The assembled measuring block was inserted into the furnace and connected to the apparatus.

In Figure 1, which shows a diagram of the measuring stand, there is no power supply connected to the heating winding of the cell module, which is switched on and off manually. Parameters such as current and voltage on the heating coil, as well as power and total energy, were recorded by the virtual instrument (VI3) described in Section 2.2.

During the preparation and assembly of the measuring block, all components included in its composition were weighed; this included measuring the same weight of water and sand. Thus, the measurements can be considered fully comparable. The only difference in subsequent experiments was the state of charge of individual cells. 

Charging and discharging of the cells was carried out using virtual devices VI1 and VI2. After performing the experiment and opening the stove, the position of the markers on the guides of the upper protective plate was checked. A photograph was taken when it was lifted. After disconnecting the measuring wires, the measuring block was disassembled and re-weighed in order to determine the mass loss of the cell, as well as the displacement of sand and possibly water between the vessels. These data can be used for energy calculations. After the mass measurements, the cell module was disassembled and a photograph of the remains inside the heat shield was taken. 

## 3. Results

The first results, related to the differences in the behavior of cells depending on the state of charge, were obtained in an experiment that involved placing three elements next to each other: a fully charged cell, a heater and a second cell discharged to the minimum voltage. A specially made heater had a cylinder shape and dimensions identical to the cells. The assembly made in this way was placed on a plastic support on a layer of quartz sand.

### 3.1. Preliminary Studies

**Figure 4:**
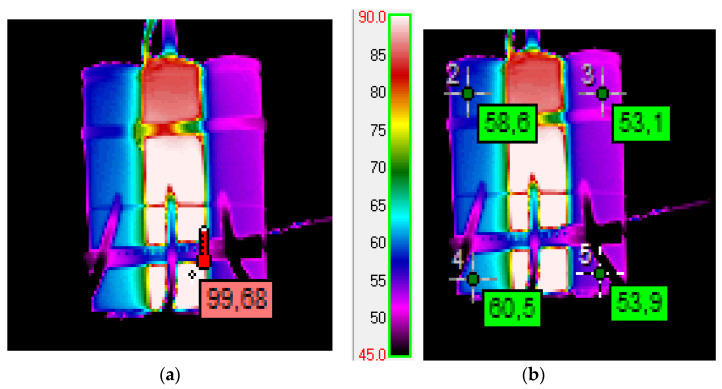
Thermogram of two cells and a heater. (**a**) Heater temperature measurement. (**b**) Comparison of cell temperatures; discharged cell on the right (results in degrees Celsius).

The preliminary tests included two cells with SOC = 100% charge: a cell on the left side and a cell with SOC = 0 on the right side (Figure 4). After switching on the heater, the temperature was recorded using RTD sensors placed on the cells and the heater. Several tests were carried out, in which the obtained results indicated a slightly faster heating rate for the charged cell. However, these results were dependent on where the sensors were placed. In order to explain the reasons for this phenomenon, thermal imaging measurements were carried out.

The condition shown in the photograph was recorded about 10 min after the heater was turned on. The thermograms show that the heater used did not have the same thermal efficiency along the entire length; therefore, the upper parts of the cells had a lower temperature. With relatively small increases in temperature from the nominal temperature and differences in the temperature of one cell, conclusions drawn regarding the dependence on the state of charge would be burdened with a large possibility of error.

The measurement results are shown in Figure 4.

The presented thermograms indicate a higher heating rate for a charged cell compared with a discharged one.

### 3.2. Examinations Using the Test Stand

The test stand did not provide the possibility of thermographic measurements. It uses a cell module that thermally insulated the cell from the environment, but the heater completely covered the cell inside the insulation; thus, the electricity supplied to the heater went, in a significant part, as heat to the cell. Due to the metallized insulating layer, the energy radiated towards the outside was reflected back towards the cell. Energy losses outside the modules were identical in each of the measured cases. The energy supplied to the cells was constant. The size of this power was a matter of choice. With heating that was too fast, there were problems with accurately distinguishing the moment of initiation of destructive phenomena. Heating that was too slow forced a long wait for the final effect. Powers of 6, 8, 9, 10, 12 and 15 W were taken into account. As a result, it was assumed that the power of 9 W would be appropriate for all experiments and this rate of energy supply was used for all experiments described in this paper.

In the presented results, the given energy value indicates the amount of energy supplied to the heater. In order to specify and estimate the energy losses that went outside, it is possible to refer to the standard, the measurement results of which are included in this paper’s Appendix A. However, this aspect is not considered in this paper. Since the mechanism of losses in all tests was identical, the differences in the results depend only on the state of charge of the cells.

**Figure 5:**
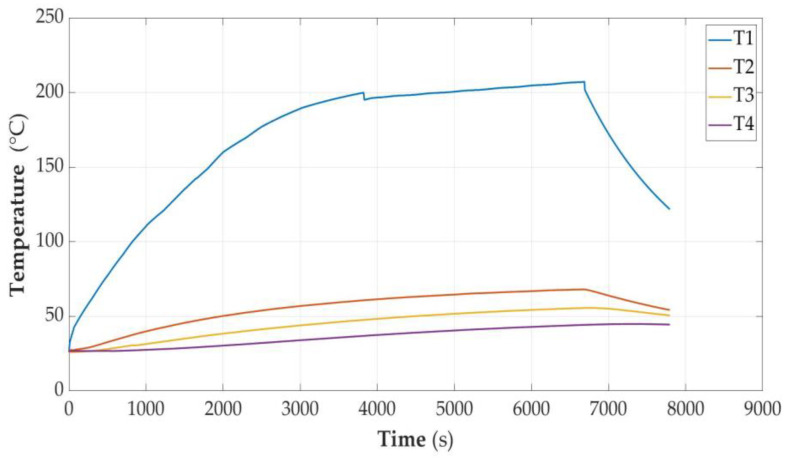
Graphs of temperature changes as a function of time *t*(s) during heating.

**Figure 6:**
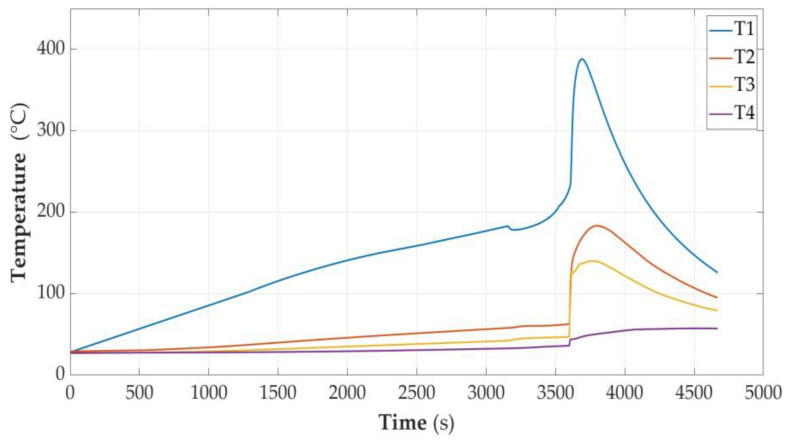
Graphs of temperature changes during heating and energy supplied to the cell module in the SOC = 30% state.

**Figure 7:**
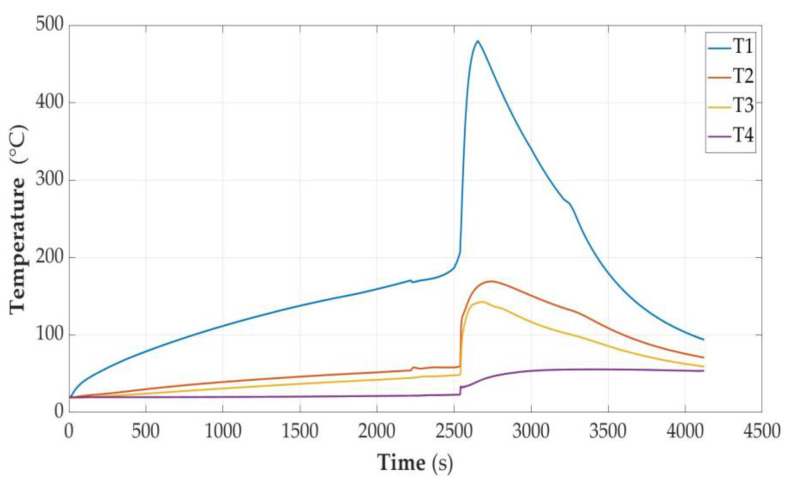
Graphs of temperature changes during heating and energy supplied to the cell module in the SOC = 50% state.

**Figure 8:**
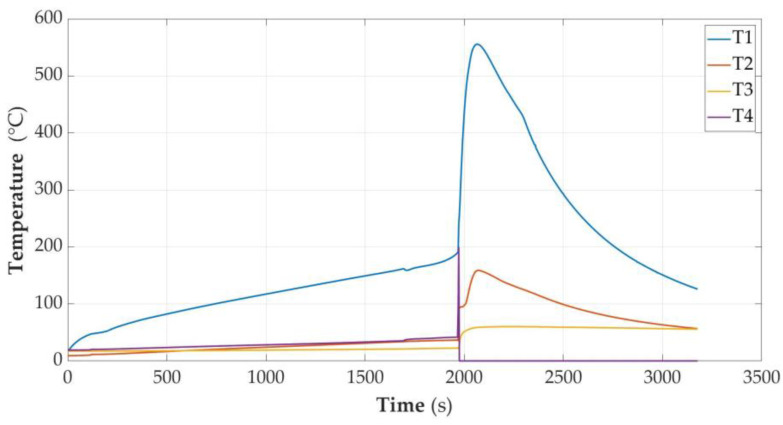
Graphs of temperature changes during heating and energy supplied to the cell module in the SOC = 100% state.

**Figure 9:**
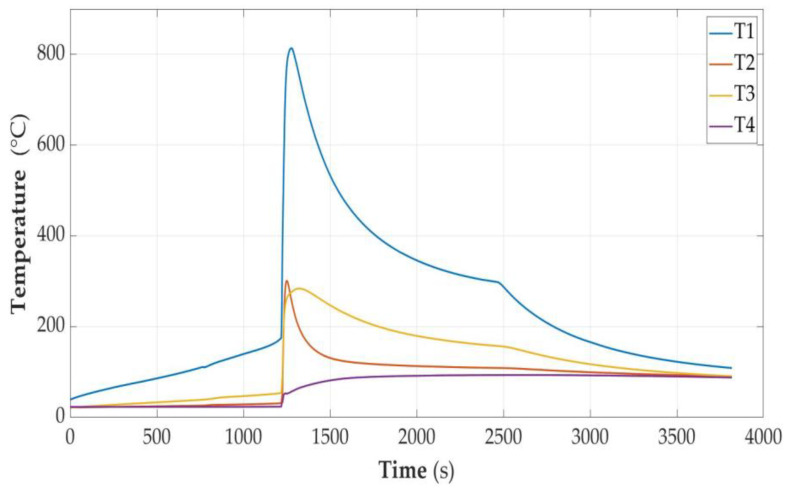
Graphs of temperature changes during heating and energy supplied to the cell module in the SOC > 100% state.

**Figure 10:**
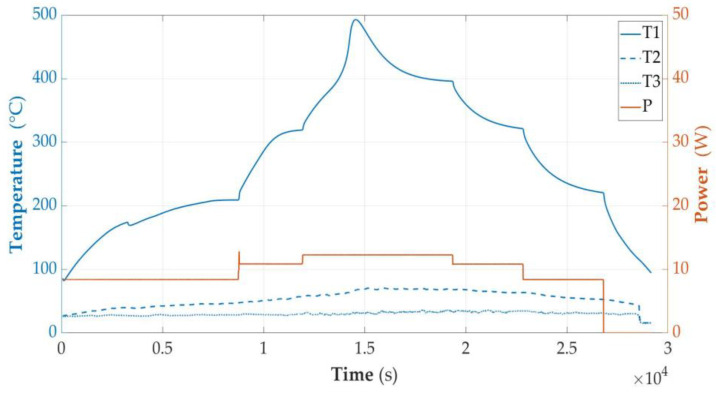
Graphs of temperature changes during heating and energy supplied to the cell module in the SOC < 0% state.

In Figure 5, Figure 6, Figure 7, Figure 8, Figure 9 and Figure 10, the following designations have been adopted: T1—temperature on the cell wall; T2—air temperature; T3—sand temperature; T4—water temperature.

#### 3.2.1. Acceptably Fully Discharged Cell

The cell discharged in an acceptable manner to SOC = 0. A fully charged cell was discharged to a voltage of 2.5 V. Leaving such a cell unconnected to the discharge load, but with the simultaneous observation of the voltage at the terminals, allows one to notice that the cell rebuilds the voltage to a value of approx. 3 V; an attempt to load such a cell does not allow it to obtain too much energy and the cell voltage decreases quickly (this phenomenon is repeatable and was observed many times in different cases).

Such a cell, when heated to 198 degrees C, was unsealed and at the same time its voltage dropped to a value slightly higher than 0.10 V; it remained in this state for a long time. This phenomenon was accompanied by a soft hiss and a slight puff of smoke. Despite heating for a total period of over 6.5 thousand seconds and heating to a temperature of over 208 degrees C, ignition did not occur. After turning off the heating, the method of cooling was observed and it was checked whether spontaneous fire would occur. After the end of the experiment and disassembly of the cell module, it was found that the cell still had the factory plastic cover, which was not discolored. The weight of the cell decreased by 2 g.

#### 3.2.2. Cell Discharged to the State of Capacity SOC = 30%

The cell was unsealed at the consumed energy of 7.98 Wh, and the temperature was 178.9 °C. This phenomenon was accompanied by a hiss and discharge of white smoke—a slightly larger amount than in the case of a discharged cell. 

After unsealing, there was a minimum temperature drop of 4 °C. Further observation allowed for the conclusion that the temperature increase was faster than would result from heating. After another 200 s, there was an avalanche increase in temperature, reaching a value of 387 °C with the total energy supplied being 9.19 Wh. After dismantling the cell module, a loss of cell weight of 37 g was detected.

#### 3.2.3. Cell Partially Discharged (SOC = 50%)

Heating until unsealing lasted 2258 s. The cell temperature was then 168.9 °C. Energy delivered was 5.62 Wh. There was a temperature avalanche, and it reached the level of 478.5 °C with the energy supplied being 6.62 Wh.

The combustion process was much more intense than in the case of a cell with SOC = 30%. The closure of the cell (weight: 1.6 g) from the positive pole side was torn off. A wad of partially burnt copper foil with a mass of 4.5 g was thrown outside. The remains of the cell shell and the contents inside the unburnt parts had a mass of 15.2 g. The unburned parts had a mass of 21.6 g and the weight loss was 45.4 g.

#### 3.2.4. Cell Fully Charged (SOC = 100%)

The cell temperature at the time of unsealing was 159.1 °C. Energy delivered was 4.29 Wh. After about 550 s, there was a temperature avalanche and it reached the level of 552 °C with the energy supplied being 5.13 Wh. Observing the course of temperature changes on the cell, it could be assumed that after unsealing, even turning off the heating would not stop the explosion due to the thermal runaway process, which had probably already started. This indicates the steepness of the course after a short-term decrease in temperature as a result of unsealing. As a result of the explosion, a large amount of gas was released from the device for several seconds, with white smoke and black streaks. Inside the measuring vessel, the temperature rose to over 200 degrees and the sand temperature jumped to about 160 °C. Unfortunately, the air temperature sensor was damaged at the time of the explosion and no data were obtained from it at the most interesting time. However, these values should be approached with caution, as a temporary temperature peak may indicate local overheating or even exposure of the sand to the stream of gases. The amount of received energy should rather be inferred a few or a dozen or so seconds after the moment of explosion.

After dismantling the cell module, a loss of cell weight of 48 g was found. Therefore, most of the initial mass (67 g) left the shell of the cell. Charred pieces of black slag were found inside the shell. In the sand, there were single small pieces of copper foil with an area of not more than 2 mm^2^.

#### 3.2.5. Overcharged Cell (SOC > 100%)

As noted earlier, such a state is operationally unacceptable and pointless. During the heating of an overcharged cell, two types of cell behavior were observed. In the first case, the cell was normally charged using the CCCV method to a voltage of 4.2 V. Charging was turned off for 6 h, during which the voltage dropped automatically to 4.17 V. Then, charging was turned on again with the current limited to 0.4 A with simultaneous observation of the voltage. The voltage increased, but at 4.93 V, further charging was turned off for fear of explosion in the charging system, which was not protected against it. After 48 h, the voltage dropped to 4.58 V and stabilized at this level. The cell was measured in this state. The cell was unsealed at a temperature of 110.3 degrees and the supplied energy of 2.19 Wh. The onset of the violent ignition phase took place at an energy input of 3.03 Wh and a temperature of 174 degrees. The peak temperature reading was 813 degrees when the supplied energy was 3.19 Wh.

After dismantling the cell module, it was found that only a metal shell with a mass of 9 g remained in the heat shield (58 g of cell mass loss). No particles of copper foil were found in the sand or in the crust. This proves its complete burnout or atomization. In addition, on the inner cover, apart from the usual black exhaust fumes, there was also a stain of copper sprayed on the glass. The position indicators of the upper protection plate showed that the plate was raised to a height of 3 mm during the measurement. The smoke coming out of the stove was gray at first and then decidedly black. The beginning of the emission of smoke was accompanied by the sound of an explosion.

The second type of behavior was observed incidentally in the previous measurement series and was not repeated in the present study. At that time, a different design of the measuring block was used, including one without an upper protective plate. Furthermore, the measuring block was not placed in the stove. The cell prepared for testing was connected to a power supply with voltage stabilization of 5 V and current limitation to 3 A, with full awareness that such charging may damage the cell. 

The goal was to obtain an overcharged cell. At the observed voltage of 4.73 V, the internal fuse was disconnected and the cell at the terminals showed a voltage of 0 V. The cell in this condition was stored for about a week before starting the tests. After connecting to the apparatus and turning on the heating with a power of 8 W, the first small explosion occurred after only a few dozen seconds with a simultaneous abnormal increase in temperature, greater than with the same power in other states of charge. The explosion was accompanied by the emission of a small amount of white smoke. For some time, the temperature decreased and then gradually increased. The thermal runaway had probably already started. It is difficult to determine its beginning, but there is a high probability it ran from a temperature of about 75 degrees, 130 s after the start of the research, with the energy absorbed by the cell module E = 0.247 Wh. At the time of about 1250 s, a violent explosion occurred, which caused both covers of the measuring block to be torn off; the cell was ejected to a height of about 2 m, with the measuring cables being broken at the same time. The cables pulled the LabView cassette and dropped it to the ground (the mass of the cassette with modules was about 0.7 kg). 

The cell, flying in the air, rapidly emitted a large amount of mostly black smoke, which also displayed gray streaks and red flames. After falling to the ground, it kept spinning for several seconds while the remnants of the material from inside burned out. The last data recorded up to the time of the eruption indicated the last measurement of the temperature of the sand at 289 degrees and the air at 377 degrees. The penultimate measurement of the cell temperature indicated 927 degrees. However, the last recorded value of 1328 degrees may have resulted from the burning of the temperature sensor wires.

The description of the course of this experiment has been quoted in order to illustrate the danger associated with misinterpretation of the voltage measurement at the cell terminals in order to check it. The value of zero can be the result of either a complete discharge or the activation of an internal fuse in an overcharged cell.

#### 3.2.6. Cell Fully Discharged (SOC < 0%)

Discharging the cell below the minimum voltage value can be carried out to a value close to zero with a small current and then by switching off the load discharging the cell. Then, after a short time, the cell partially regenerates and again shows a voltage close to 2–3 V. From an energy point of view, the efficiency of a cell rebuilt in this way is very low. Thermal tests of such a cell brought results very similar to those in the case of a cell discharged to the minimum permissible value (as in Section 3.2.1); therefore, they are not described again here.

However, it is possible to discharge to 0 V and then leave the cell with a low-resistance load attached for a longer time, counted in days. In this case, the cell, even after removing the short circuit, shows a very low voltage, in a range from 0.1 V to 0.5 V or 0 V. The course of heating and temperature measurements of a cell discharged in this way are shown in Figure 10.

Figure 10 shows the following: T1—temperature on the cell wall; T2—air temperature; T3—sand temperature; and P—power supplied to the system. In this case, however, the heating power was not kept constant as in the tests described in Section 3.2.1, Section 3.2.2, Section 3.2.3, Section 3.2.4, Section 3.2.5 and Section 3.2.6, but the heating of the cell was started with a heating power of 8.5 W, and then it was increased to 11 W and 12 W when a longer period of temperature stabilization was observed. At the power of 8.5 W, the results were again similar to those in Section 3.2.1 and the temperature was stabilized at 208 degrees and for about 9000 s. After increasing the power to 11 W, the temperature was raised to about 330 degrees and stabilized again. The phenomenon of temperature increase was accompanied by burning of the contents, but after 12 thousand seconds there was another stabilization. This was interrupted by another increase in the heating power to 12.5 W, which caused temperature to increase, at first gently and then in a way that indicated the burning of the contents and the supply of energy from inside. As a result, a temperature of 490 degrees was reached, which, after the cell content burned out, began to decrease automatically, despite maintaining the heating power at 12.5 W. This experiment, in turn, shows that even a completely discharged cell can be a combustible material. If a sufficiently large amount of energy is transferred from the outside, such a cell is a fuel.

It can be said that for the full burnout of the cell, it was necessary to use additional energy of over 28 Wh, and with the heating mode used, burnout took over 14,000 s.

#### 3.2.7. Temperature of Cell as a Function of Energy (E) Supplied to the Cell Module

**Figure 11:**
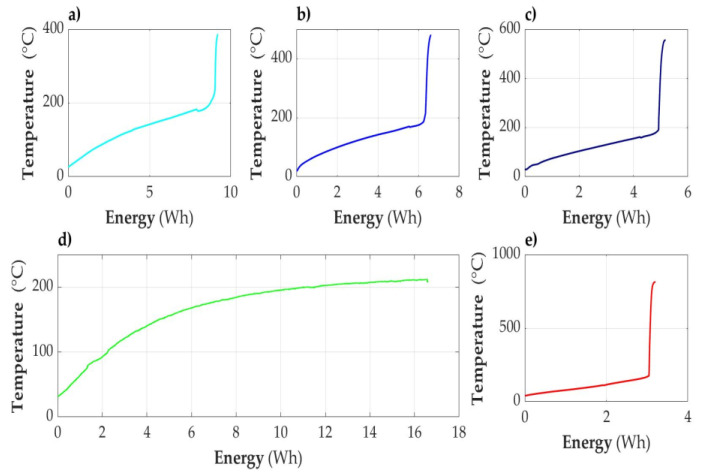
The dependence of the cell temperature on the energy supplied to the cell module. (Energy supply power—constant and equal to 9 W for all cells). (**a**) Cell partially discharged (SOC = 30%). (**b**) Cell partially discharged (SOC = 50%). (**c**) Cell fully charged (SOC = 100%). (**d**) Unloaded cell (SOC = 0%). (**e**) Cell overcharged (SOC > 100%).

The energy (E) supplied to the cell module can be described as a function of time (t), E = f(t), and similarly, the temperature (T) on the cell surface is dependent on time; T = g(t). Therefore, time can be treated as a parameter and the relationship T = h(E) can be formulated. This operation was carried out on experimental data for each of the sets of temperature results from successive experiments with cells with different states of charge (SOCs). The range of input data was limited to the moment when the temperature on the surface of the cell reached its maximum as a result of the combustion processes of the material inside. These relationships are shown in Figure 11. At the same time, each of the graphs indicates with its top the maximum temperature reached by the cell after ignition.

**Figure 12:**
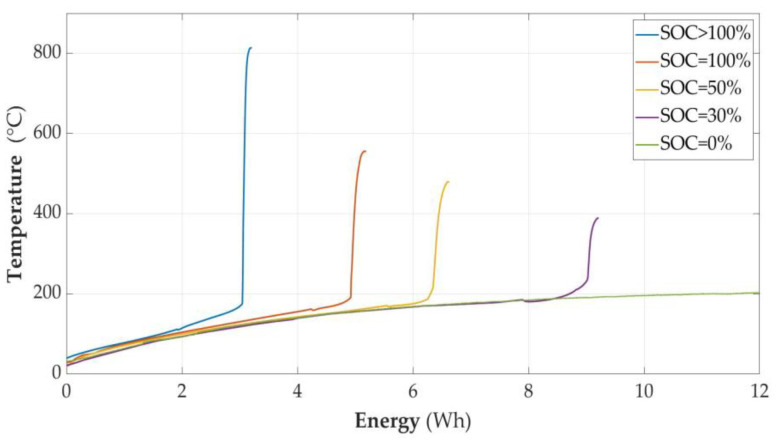
Comparison of flammability properties of cells with different states of charge as a function of Energy supplied to the cell module.

In order to visualize the differences, the axes were standardized and all graphs shown in Figure 11 were collected simultaneously in Figure 12.

This figure illustrates the fact that the energy supplied to the cell module that causes its avalanche fire is strictly dependent on the state of charge of the cell; this is the key statement of this work. In all the cases involving charged cells, during the supply of energy to them, there was an unsealing and, as a result, gas leakage. 

In all the cases involving charged cells, during the supply of energy to them, there was an unsealing and, as a result, gas leakage. The next phase of the burning process consisted in firing up the interior, and the following phase was a violent explosion combined with the ejection of some of the cell contents outside.

#### 3.2.8. Estimation of the Amount of Energy Released by the Cell in the Developed Phase of Thermal Runaway

An interesting aspect of the research that was carried out is the estimation of the amount of energy released by the cell in the developed phase of thermal runaway. The sum of the increase in the amount of heat in the system in the time between the state at the beginning of the avalanche temperature increase and the moment when the maximum is followed by a decrease in water temperature was adopted as the measure of the released energy. This time is relatively long and amounts to more than a few minutes. 

The choice of such an end point is justified by the fact that at this moment, the thermal conditions are leveled to such an extent that the inner vessel with sand and cell residues gives off less energy than the intermediate vessel loses. During the tests, the masses of sand, water, vessels, lids and cells were measured. The authors of the research are aware that, unfortunately, estimating the accuracy of energy determination in this way is very difficult, but in the current state of research, they do not have the equipment to obtain better metrological parameters.

The basic error that appeared in the measurements was unsealing of the vessels during the measurements at SOC = 100% and at SOC > 100%. Some of the hot exhaust gases rushed out of the measuring vessels, carrying energy with them. Therefore, the obtained results should be treated as a lower estimate and it is true that the energy released during the explosion was not less than the calculated E_sum_ (Wh) value, which is a conversion of the heat calculated according to the rules given below. In addition, there was no guarantee that the entire volume of sand was at the same temperature, but assuming a relatively long time between the start of the avalanche and the end of the heat transfer, the time allowed the temperature to even out. Another error that cannot be resolved involves determining the temperature of the vessel walls above the water level. It was assumed that it was the same as the temperature of the water for the outer vessel, and the same as the temperature of the air above the sand for the inner vessel. The lower part of the inner vessel was assumed to be at the temperature of the water. The temperature of the lid was assumed to be equal to the temperature of the air inside the inner vessel. Determining the mass of the cell is also problematic because the cell disintegrates after the explosion and the heat from its parts passes to the sand, hence only the mass of the residue measured during unpacking after the measurement of the measuring module was taken as the mass of the cell module after the explosion. Using the above assumptions, the amount of released heat can be defined as in Formula (1).

(1) $$Q_{sum}=Q_{sand}+Q_{wat}+Q_{air}+Q_{cov}+Q_{inl}+Q_{inu}+Q_{imv}+Q_{mcell}$$

where, respectively, the terms of the equation denote the heat stored

*Q_sand_*—in the sand;
*Q_wat_*—in the water;
*Q_air_*—in the air contained in the inner vessel;
*Q_cov_*—in the cover (outer cover);
*Q_inl_*—at the bottom of the inner vessel;
*Q_inu_*—in the upper part of the inner vessel;
*Q_imv_*—in an intermediate vessel;
*Q_mcell_*—in the remains of the link.
*Q_sum_*—Total heat equivalent to the energy released during the combustion of the cell.
*E_sum_* -Total energy resulting from the conversion of heat *Q_sum_* in J to Wh.

Each term of Formula (1) was calculated according to the basic Formula (2):

(2) $$Q_{i}=m_{i}*h_{i}*\left(T_{hi}-T_{li}\right)$$

where

*m_i_*—is the mass of the component;
*T_li_*—is the temperature at the beginning of the avalanche of a given component;
*T_hi_*—is the temperature at the end of energy transfer;
*h_i_*—is the specific heat of component *i*.

For all components, the initial moment of the measured energy state is the same, as is the final moment. For obvious reasons, the initial and final temperatures are different, taking into account the assumptions made above.

The temperature measurement values were taken from the time characteristics of temperature changes as in Figure 6, Figure 7, Figure 8 and Figure 9, but for higher accuracy they were read directly from data files. The values of the specific heat of the parameters were taken from the material tables [33] and are presented in Table 2. For the remainder of the cell, the specific heat of the heat-resistant fabric was assumed.

The results of calculations of individual components of heat and energy released during the thermal runaway process in various SOCs are presented in Table 3.

## 4. Discussion

**Figure A1:**
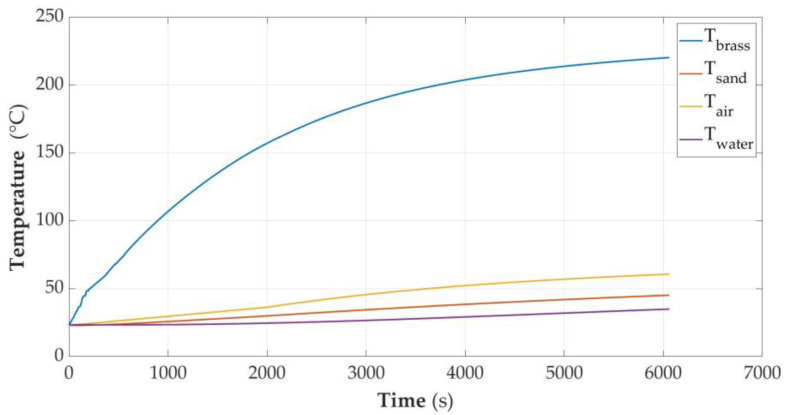
The dependence of the brass model temperature on the energy supplied to the cell module. (Energy supply power: constant and equal to 9 W).

The results obtained during the tests can be grouped as follows:

- It was experimentally proven that both the ignition parameters and the course of the cell fire depend on the state of charge, as shown in Table 4. The results related to the time of heating with constant power were characterized in the function delivered to the energy cell module. According to the authors, this is a more universal form of presentation. Another result is that overcharging the cell makes it much more susceptible to ignition, and the fire of this cell is much more violent. The temperature reached is of the order of 813 degrees Celsius. The measured parameters of the unsealing point as a function of Energy are also presented in Table 4.
- This table also contains a summary of the maximum temperatures that occurred on the cell wall during the measurements. However, it can be assumed that the temperature that occurred at the outlet of the cell in the flue gas stream was even higher due to the traces of sputtered copper on the glass cover described in this work and the lack of copper in the remains of the burnt cell in the case where the SOC = 100% and the overcharged case.
- It was found that the higher the charge level, the less energy triggers the ignition of the cell. It is worth paying attention to the fact that the temperature differences at which the cells were unsealed were not very large, but the amount of energy causing the unsealing differed significantly. As mentioned before, the energy shown in the graphs is not exactly the value delivered to the cell, but the value delivered to the cell module. Therefore, it represents the energy supplied to the cell plus the energy losses in the elements included in the cell module. Despite this simplification, due to the repeatability of the experiments, it is possible to state that the amount of energy supplied to the cell causing its unsealing depends on the state of charge of the cell.
- Graphs of cell temperature changes over time were obtained with high accuracy and resolution, at different SOCs.
- The results of temperature measurements as a function of time were brought to dependence on the energy supplied to the cell. See Section 3.2.7.
- The amount of energy released from the cell during the thermal runaway process was also estimated at different SOCs. See Section 3.2.8 Table 3.
- A graph of temperature versus time was obtained for a model of a cell made of brass and placed in a cell module. Measurements were carried out under identical conditions to those in Section 3.2.1, Section 3.2.2, Section 3.2.3, Section 3.2.4, Section 3.2.5 and Section 3.2.6. In order to find a pattern enabling estimation of the size of external losses, a model of a brass cell was made, reflecting its shape and dimensions (the weight of the model was 176 g), and then heating was carried out as in the experiments described in Chapter 3. These calculations have not been performed, but the data may be helpful in the process of creating a simulation model. Temperature change graphs are shown in Figure A1 in Appendix A.
- Organoleptic descriptions of the cell residues at various SOC states were obtained. This is significant knowledge that is useful during post-fire tests, as it allows for a rough assessment of the state of charge of a burnt-out cell. This knowledge may be the basic premise in the event that the data recording system is also destroyed. In addition, current BMSs control the cell chains without recording parameters with an accuracy of one cell.
- The presented results were measured with a time resolution of 3 s of recorded data. However, they were measured every 0.1 ms and averaged. Measurement resolution in each channel was 24 bits, as in Section 3.2.1, Section 3.2.2, Section 3.2.3, Section 3.2.4, Section 3.2.5 and Section 3.2.6. The problem, however, was the escape of part of the flue gas in the tests for SOC = 100% and over 100%. In these cases, the estimates of the released energy are probably higher than those calculated and shown in Table 3. As has already been indicated in the text describing the method of estimating the amount of energy released, the assumption made about the temperature measured at points and that it applies to the entire volume of the medium is a significant simplification, despite even the relatively long time for temperature equalization. An acceptable solution is to create a simulation model for which the conducted tests can be input as data.

## 5. Conclusions

Knowledge of the differences in the behavior of cells with different states of charge may be important in the construction of safer batteries and, consequently, energy storages. 

The intention of the authors of this paper is to build a simulation model that allows mapping of the obtained experimental data in the first stage. The next intended step is a simulation that allows presentation of the energy flows in the battery in the cases of various states of charge, and observation of the fire hazard. In the case of repeating the experiment, e.g., for a different type of battery, it would be advisable to modernize the measuring station in such a way as to better capture energy from exhaust gases, in particular in experiments in which SOC > 50%.

Knowledge of the phenomena during the triggering of a cell fire by means of externally supplied thermal energy may be a step to explaining why some battery fires occur even though the batteries were disconnected from the control system. The problem will be particularly acute when energy storages made of recycled cells start to be built en masse. For these batteries, obtaining the identity of the component cells will be more difficult than in the case of assembly from new cells, which in turn will result in the need to modernize BMSs.

The amount of energy absorbed by the cell in various forms in conjunction with the SOC can be a good parameter signaling the state of danger in the cell. The observation of the worst flammability properties of partially discharged cells can be used to build BMS control algorithms that increase the battery’s resistance to fire. For this purpose, after detecting a cell with deteriorated properties in the battery, one should strive for zonal discharge of the surrounding cells adjacent to the damaged cell. In this way, even if a fire-causing cell is ignited, its spread will be slowed down by a possible secondary fire of cells with a lower SOC. Such a procedure is particularly recommended in large energy storage facilities where cells with deteriorated parameters are used. However, this requires BMS supervision over each cell individually, instead of over groups of cells with theoretically identical parameters connected in parallel. This is another issue requiring research related to the control algorithms built into battery management systems and thermal management systems.

More difficult-to-consider indications resulting from the current work are the role and mode of operation of the cell’s internal fuse. The fact that the cells have an internal fuse that disconnects them from the external circuit is potentially dangerous. Naturally, with an external short circuit, this fuse is necessary. However, its operation causes a voltage equal to zero at the cell terminals and such a cell can be particularly dangerous due to the charge remaining in it. The cells should either have an indicator of the amount of charge inside each cell, independent of the terminals, which would only be an indicator, or an additional electrode that allows a cell to be discharged in the event of a fuse trip. Such an electrode, operating once, could reduce the charge in the cell to zero and thus make it safe. The role of such cell electrodes would also be significant in the event of car accidents. After the main battery power supply is cut off after an accident, it would be possible to automatically slowly discharge the cells through additional safety circuits, which would reduce the occurrence of fires in cars involved in accidents, or in energy storages. However, this requires the involvement of cell manufacturers.

The results presented in this work can also be a basis for improving battery simulation programs and their behavior in extreme conditions, including damage conditions.

## Figures and Tables

**Table 1:** Parameters of the cell used for testing.

Model:	INR21700-40T (40T)
Size:	21,700
Style:	Flat Top
Nominal Capacity:	4000 mAh
Discharge Current:	35 A Maximum Continuous (1C)
Nominal Voltage:	3.6 V
Maximum Voltage:	4.2 V
Cutoff Voltage:	2.5 V
Approximate Dimensions:	21.1 mm × 70.4 mm
Approximate Weight:	66.8 g
Detailed Specification Datasheet:	Samsung 40T Datasheet [32]

**Table 2:** Values of specific heat of substances present in the measuring block in (J/(kg*K).

Sand	Water	Air	Steel	Glass	Cell Module Remains
800	4145	1008	478	740	840

**Table 3:** Calculation results of the energy released during the thermal escape process. *Q_i_* expressed in (J), total energy in (Wh).

SOC	*Q_sand_*	*Q_wat_*	*Q_air_*	*Q_cov_*	*Q_inl_*	*Q_inu_*	*Q_imv_*	*Q_mcell_*	*Q_sum_*	*E_sum_*
(%)	(J)	(J)	(J)	(J)	(J)	(J)	(J)	(J)	(J)	(Wh)
30	3925	13,866	39	3037	1237	753	1542	7138	31,537	8.76
50	9240	19,832	103	8026	496	753	1991	7356	47,797	13.27
100	12,283	19,747	139	10,850	1372	2691	1982	18,091	67,156	18.65
>100	9482	49,156	171	12,497	1512	3307	4935	15,421	96,483	26.79

**Table 4:** List of ignition parameters of cells in different states of charge.

	Unsealing of the Cell		Beginning of Avalanche		Maximum Temperature
	Energy (Wh)	Temperature (°C)	Energy (Wh)	Temperature (°C)	Temperature (°C)
SOC > 100%	1.99	113.2	3.03	171.2	813.7
SOC = 100%	4.29	159.1	4.90	187.2	552.0
SOC = 50%	5.62	168.9	6.23	187.9	478.5
SOC = 30%	7.98	178.9	8.95	211.1	387.6
SOC = 0%	9.95	194.2	--	--	--

## Data Availability

The “raw” measurement datasets have not been made publicly available. All the data necessary to prove the theses of the work have been presented in the form of graphs.
